# Profiles of depressive symptoms and influential factors among people living with HIV in China

**DOI:** 10.1186/s12889-023-15057-4

**Published:** 2023-01-23

**Authors:** Dongfang Wang, Qijian Deng, Huilin Chen, Min Wang, Zhening Liu, Honghong Wang, Xuan Ouyang

**Affiliations:** 1 Department of Psychiatry, National Clinical Research Center on Mental Disorders, the Second Xiangya Hospital of Central South University, Changsha, China; 2 National Technology Institute On Mental Disorders, the Second Xiangya Hospital of Central South University, Changsha, China; 3 School of Psychology, Centre for Studies of Psychological Applications, Guangdong Key Laboratory of Mental Health and Cognitive Science, Ministry of Education Key Laboratory of Brain Cognition and Educational Science, South China Normal University, Guangzhou, China; 4 Department of Psychiatry, University of Oxford, Oxford, UK; 5 Institute for HIV/AIDS, the First Hospital of Changsha, Changsha, China; 6 Xiangya Nursing School, Central South University, Changsha, China

**Keywords:** Depressive symptoms, People living with HIV, Family function, Resilience, Childhood trauma

## Abstract

**Background:**

Depressive symptoms are highly prevalent among people living with HIV (PLWH). We leveraged Latent Profile Analysis (LPA) to identify profiles of depressive symptoms among PLWHs. We also investigated differences in psychological factors of interest, demographic characteristics, and HIV-related factors across patients’ profiles.

**Methods:**

A cross-sectional study was conducted at one hospital and two designated prison facilities in Hunan province, China. A total sample of 533 PLWHs (320 recruited from the hospital, 213 recruited from prisons) completed the survey. Depressive symptoms were assessed using the 9-item Patient Health Questionnaire (PHQ-9). Family function, resilience, childhood trauma, demographic characteristics, and HIV-related factors were also evaluated. We conducted LPA and multinomial logistic regression analyses to: 1) identify distinct profiles for depressive symptoms; 2) identify demographic characteristics, and HIV-related, and psychological factors predicting PLWHs’ likelihood to express a specific profile.

**Results:**

We identified three distinct profiles of depressive symptoms among PLWHs: severe symptoms (11.8%), moderate symptoms (40.5%), and low/no symptoms (47.7%). Moderate/ severe family dysfunction, low resilience, experiencing emotional abuse and neglect were more likely to fall in the “severe symptoms” rather than the “low/no symptoms” profile. In addition, severe family dysfunction, low resilience, and experiencing emotional neglect indicated a higher likelihood of being classified in the “moderate symptoms” profile, compared to the “low/no symptoms” profile.

**Conclusion:**

Identifying profiles of depressive symptoms among PLWHs using the PHQ-9 items allows for understanding of the distinct paths of development of depressive symptoms and for developing tailored prevention and intervention programs for PLWHs.

## Background

As is evident, depression is the most common mental disorder among people living with HIV/AIDs (PLWH), the prevalence of which is estimated to be two to four times higher than in general population [1, 2]. For instance, Wang and colleagues reported that prevalence of clinical depression or depressive symptoms (which do not necessarily meet the entire diagnostic criteria for a depressive disorder) in general PLWH to be approximately 50.8% in China [3]. Meanwhile, depressive symptoms have been identified as a risk factor against broader health outcomes among the PLWH, such as reducing one’s antiretroviral therapy adherence, increasing HIV viral loads and lowering CD4 counts [4, 5], which result in impaired immunological response and even heightened mortality [6, 7]. As such, screening for depressive symptoms addresses an overriding concern in identifying risk factors for adverse health outcomes among PLWHs.

Although depressive symptoms have been widely reported in PLWHs around the world, most studies are based on variable-oriented approaches that fail to reveal different patterns between individuals and may therefore draw over-generalized conclusions based on overall samples [8]. In contrast, person-oriented approaches capture information at the individual level, allowing for a more fine-grained understanding of symptom presentations, ideal for psychopathology research [9]. The uses of person-oriented approaches, such as latent class analysis (LCA), latent profile analysis (LPA), to explore the structure of psychopathology have become increasingly popular over the past decade. LPA is a type of LCA that uses continuous indicators of symptom severity rather than binary indicators (i.e., symptom absence or presence [10]), which can provide assessment of symptom profiles with greater granularity.

In recent years, a growing number of studies employed LPA to explore the profiles of depressive symptoms in clinical [11, 12] and non-clinical samples [13, 14]. For instance, Saracino et al. (2020) identified a four-class solution among patients with cancer, which were termed as the “no/low symptoms” group, “mild depressive symptoms” group, “patterned response” group, and “moderate depressive symptoms” group [11]. Moussavi et al. (2021) applied LPA to discern heterogeneous patterns of anxiety and depressive symptoms among youth in foster care, and confirmed the presence of three subtypes: low, medium, and high symptom profile [15]. However, the heterogeneity of depressive symptoms among PLWHs has only been scarcely studied.

Collectively, we carried out a mental health screening survey for PLWHs in Hunan province in China with the support of the local government, which offered an opportunity to explore the heterogeneity of depressive symptoms among PLWHs, using LPA. Much literature suggests that individual characteristics (e.g., sex [16], resilience [17]), environmental factors (e.g., family function [18]), and previous life events (e.g., childhood trauma [19]) all have significant effects on depressive symptoms. Thus, the present study aimed to further explore whether relevant factors (e.g., socio-demographics, family function, resilience, and childhood trauma) are significant predictors of distinct profiles of depressive symptoms in PLWHs. Based on previous work, we speculated that LPA would identify several different depressive symptom profiles among PLWHs (Hypothesis 1). We also anticipated that resilience, family function, and childhood trauma were significant predictors of distinct profiles for depressive symptoms (Hypothesis 2).

## Materials and method

### Participants and procedure

The participants were 533 PLWHs from one hospital and two designated prison facilities in Hunan, China. A convenience sample of 320 PLWHs who regularly visit the hospital was recruited from HIV/AIDS clinic of the First Hospital of Changsha (Sampling time: from March 2019 to June 2019), and the participants were all outpatients. Meanwhile, in August to September 2019, a cluster sampling of participants from two prisons dedicated to the incarceration of HIV-infected prisoners was conducted, and data from a total of 213 valid samples were obtained. The survey was conducted voluntarily and anonymously, and all participants (or their caregivers, if age < 18) signed an informed consent form before starting the survey. Participants can withdraw from the study at any time if they feel uncomfortable. Detailed sampling and data collection have been described in elsewhere [20].

This study was carried out in accordance with the Helsinki Declaration as revised 1989 and approved by the Ethics Committees of Xiangya Nursing School of Central South University (No.2018007).

### Measures

#### Demographic characteristics and HIV-related factors

Demographic characteristics and HIV-related factors included: participant source [1 = prisoners, 2 = outpatients], sex [1 = male, 2 = female], age, education level (year), duration of HIV infection (months), ethnicity [1 = Han (the major ethnic group in China), 2 = others], marital status [1 = unmarried, 2 = married], residence location [1 = urban, 2 = rural], route of infection [1 = sexuality, 2 = blood transfusion, 3 = needle sharing/ take drugs, 4 = unknown].

#### Depressive symptoms

The 9-item Patient Health Questionnaire (PHQ-9) was used to measure depressive symptoms [21]. Each item was rated within a time-frame of 2 weeks on a four-point Likert scale, from 0 (‘not at all’) to 3 (‘nearly every day’). Higher total scores indicate more severe depressive symptoms. The PHQ-9 has demonstrated acceptable psychometric properties in the PLWH population [22, 23], and the questionnaire is widely used in studies of Chinese PLWHs [20, 24]. In the present study, the Cronbach’s α was 0.92.

#### Family function

Family APGAR Index was used to assess family function [25]. It consists of 5 items, clustering into five dimensions: Adaptation, Partnership, Growth, Affection, and Resolve. Each item rated on a three-point Likert scale from 0 (‘almost always’) to 2 (‘hardly ever”’), with higher total scores suggesting better family function. The cut-off points for the family function scores were defined as: a total score of 0–3 reflects severe family dysfunction, 4–6 moderate family dysfunction, and 7–10 good family function. The Family APGAR Index has been widely applied in China with great validity and reliability (Cronbach’s α = 0.90) [26]. In this study, the Cronbach’s α was 0.87.

#### Resilience

The 10-item Connor-Davidson Resilience Scale (CD-RISC-10) was used to assess the degree of resilience [27]. Respondents rated each item on a five-point scale, from 0 (‘not true at all’) to 4 (‘true nearly all of the time’), and higher total scores indicate stronger level of resilience. The Chinese version of CD-RISC-10 has demonstrated acceptable psychometric properties in the clinical and non-clinical population (Cronbach’s α = 0.90 ~ 0.92) [28, 29]. In the current study, the Cronbach’s α was 0.96. In line with the classification of continuous variables in previous studies [30, 31], resilience was recoded into three categories by the 27th and 73rd percentile (scores falling at 27th percentile or below suggests poor resilience, scores between 27th to 73rd percentile suggests medium resilience, while scores falling at 73rd percentile and above suggests high resilience).

#### Childhood trauma

The Childhood Trauma Questionnaire (CTQ) was used to assess self-reported experiences of childhood trauma before age of 16 [32]. It consists of 28 items, clustering into five dimensions: emotional abuse, physical abuse, sexual abuse, emotional neglect, and physical neglect. Respondents rated each item on a five-point scale, from 1 (‘never’) to 5 (‘always’), and higher CTQ scores reflect a greater number of childhood traumas experienced. The cut-off scores for severe trauma exposure scores were defined as: emotional abuse ≥ 13, physical abuse ≥ 10, sexual abuse ≥ 8, emotional neglect ≥ 15, and physical neglect ≥ 10. The CTQ has satisfactory reliability in the Chinese clinical and non-clinical samples, with Cronbach’s α being 0.81 and 0.79 [33]. In this study, the Cronbach's α for the CTQ was 0.77.

### Statistical analysis

The LPA was conducted using Mplus 7.30 to identify profiles of PLWHs based on depressive symptoms. We analyzed whether there was a heterogeneous latent category in the PLWHs’ depressive symptoms. The fit of four models (one-, two-, three-, and four class models) was assessed subsequently. In this study, lower Akaike Information Criteria (AIC) [34], Bayesian Information Criterion (BIC) [35], sample size adjusted BIC (a-BIC) [36], higher Entropy values [37], significant outcomes from Vuong-Lo-Mendell-Rubin Likelihood Ratio Test (VLMR-LRT) and Bootstrap Likelihood Ratio Test (BLRT) [38] were indicative of a better fit of the model to the data.

Meanwhile, analyses of descriptive statistics were conducted to illustrate the demographic variables and HIV-related factors of participants using SPSS 23.0. A univariate analysis (chi-square analyses for categorical variables and independent sample t-test for continuous variables) was conducted to explore the significant associations between sample characteristics (demographic characteristics, HIV-related factors, family function, resilience, and childhood trauma) and profiles of depressive symptoms. Multinomial logistic regression was used to measure the association between profile membership and the sample characteristics. Consistent with previous work [39, 40], only significant variables in the univariate analysis were used as candidates for multinomial logistic regression analyses in the presents study to reduce the number of insignificant variables in the multivariate model. The odds ratio (OR) with a 95% confidence interval (CI) were used to estimate strengths of associations. A two-tailed *P* < 0.05 was considered statistically significant.

## Results

### Description of the sample

We included 533 PLWHs with 320 outpatients (60.0%) and 213 prisoners (40.0%) in the final analysis. The proportion of male was overwhelmingly higher than that of female (90.8% *vs.* 9.2%). The age of the current PLWH sample ranged from 15.0 to 68.0 years; the mean (SD) age was 33.01 (SD = 9.93) years. Participants’ average duration of HIV infection was 62.31 (SD = 49.68) months. Detail demographic characteristics and HIV related factors are displayed in Table 1.

**Table 1:** Comparisons of sample characteristics among profiles of depressive symptoms

Variables			Total *N* = 533	Severe disturbance, *N* = 63, 11.8%	Moderate disturbance, *N* = 216, 40.5%	High resistance, *N* = 254, 47.7%	F/χ^2^	p
Participant source [N(%)]		Prisoners	213(40.0)	40(63.5)	86(39.8)	87(34.3)	χ^2^ = 17.99	< 0.001
		Outpatients	320(60.0)	23(36.5)	130(60.2)	167(65.7)		
Sex, [N(%)]		Male	484(90.8)	58(92.1)	198(91.7)	228(89.8)	χ^2^ = 0.64	0.726
		Female	49(9.2)	5(7.9)	18(8.3)	26(10.2)		
Age (years),		M(SD)	33.01(9.93)	36.93(9.72)	33.41(10.30)	31.70(9.40)	F = 7.17	0.001
Education (years),		M(SD)	12.41(3.82)	11.21(3.62)	12.11(3.77)	12.97(3.83)	F = 6.32	0.002
Duration of HIV infection (months)		M(SD)	62.31(49.68)	79.61(59.33)	65.10(51.20)	55.60(44.41)	F = 6.01	0.003
Ethnicity, [N(%)]		Han	489(91.7)	58(92.1)	202(93.5)	229(90.2)	χ^2^ = 1.75	0.417
		Others	44(8.3)	5(7.9)	14(6.5)	25(9.8)		
Marital status, [N(%)]		Unmarried	352(66.0)	35(55.6)	142(65.7)	175(68.9)	χ^2^ = 4.02	0.134
		Married	181(34.0)	28(44.4)	74(34.3)	79(31.1)		
Residence location, [N(%)]		Urban	330(61.9)	41(65.1)	145(67.1)	144(56.7)	χ^2^ = 5.69	0.058
		Rural	203(38.1)	22(34.9)	71(32.9)	110(43.3)		
Route of infection, [N(%)]		Sexuality	287(53.8)	28(44.4)	121(56.0)	138(54.3)	χ^2^ = 12.59	0.050
		Blood transfusion	8(1.5)	1(1.6)	4(1.9)	3(1.2)		
		Needle sharing/ take drugs	76(14.3)	18(28.6)	26(12.0)	32(12.6)		
		Unknown	162(30.4)	16(25.4)	65(30.1)	81(31.9)		
Family function, [N(%)]		Good	231(43.3)	23(36.5)	76(35.2)	132(52.0)	χ^2^ = 41.14	< 0.001
		Moderate dysfunction	203(38.1)	14(22.2)	106(49.1)	83(32.7)		
		Severe dysfunction	99(18.6)	26(41.3)	34(15.7)	39(15.4)		
Resilience, [N(%)]		High	127(23.8)	4(6.3)	18(8.3)	105(41.3)	χ^2^ = 98.88	< 0.001
		Medium	254(47.7)	25(39.7)	122(56.5)	107(42.1)		
		Low	152(28.5)	34(54.0)	76(35.2)	42(16.5)		
Childhood trauma	Emotional abuse	No	501(94.0)	50(79.4)	251(92.6)	251(98.8)	χ^2^ = 35.12	< 0.001
		Yes	32(6.0)	13(20.6)	16(7.4)	3(1.2)		
	Physical abuse	No	475(89.1)	49(77.8)	186(86.1)	240(94.5)	χ^2^ = 17.92	< 0.001
		Yes	58(10.9)	14(22.2)	30(13.9)	14(5.5)		
	Sexual abuse	No	396(74.3)	36(57.1)	152(70.4)	208(81.9)	χ^2^ = 19.12	< 0.001
		Yes	137(25.7)	27(42.9)	64(29.6)	46(18.1)		
	Emotional neglect	No	341(64.0)	33(52.4)	130(60.2)	178(70.1)	χ^2^ = 9.12	0.010
		Yes	192(36.0)	30(47.6)	86(39.8)	76(29.9)		
	Physical neglect	No	267(50.1)	21(33.3)	94(43.5)	152(59.8)	χ^2^ = 20.47	< 0.001
		Yes	266(49.9)	42(66.7)	122(56.5)	102(40.2)		
	Any	No	194(36.4)	11(17.5)	66(30.6)	117(46.1)	χ^2^ = 23.19	< 0.001
		Yes	339(63.6)	52(82.5)	150(69.4)	137(53.9)		

### Model fitting

We assessed the fit of four LPA models and the statistical fit indices are shown in Table 2. The VLMR-LRT was statistically significant for models two and three. Also, the fit of the three-latent profile solution was better than a two-latent profile model, as indicated by the lower AIC and BIC values and higher Entropy values. Although the Entropy value remained highest (0.95) for two latent profiles, the choice of the three- profile model was based on the research finding that the LMR-LRT outperformed all other tests available for identifying the correct number of classes in mixture modeling [41]. Therefore, the three-profile solution was selected as the optimal model. Additionally, the predictive model was acceptable, with positive predictive values ranging from 95 to 98%.

**Table 2:** Fit indices for latent profile analyses

Class	BIC	aBIC	AIC	BLRT (p)	Entropy	aLMR (p)	VLMR (p)	Smallest class (%)
1	12,870.02	12,812.88	12,793.01					
2	10,934.41	10,845.53	10,814.61	< 0.001	0.95	< 0.001	< 0.001	26.3
**3**	**10,264.44**	**10,143.82**	**10,101.86**	**< 0.001**	**0.91**	**< 0.001**	**< 0.001**	**11.8**
4	10,105.90	9963.54	9900.53	< 0.001	0.89	0.427	0.422	6.4

Bold indicates best fit. Entropy and value of p for BLRT, aLMR, and VLMR are not applicable to a one-class solution

*BIC* Bayesian information criterion, *aBIC* Sample-size-adjusted Bayesian information criterion, *AIC* Akaike information criterion, *BLRT* Bootstrap likelihood ratio test, *aLMR* Adjusted Lo-Mendell-Rubin, *VLMR* Vuong-Lo-Mendell-Rubin

### Class assignments

**Fig. 1:**
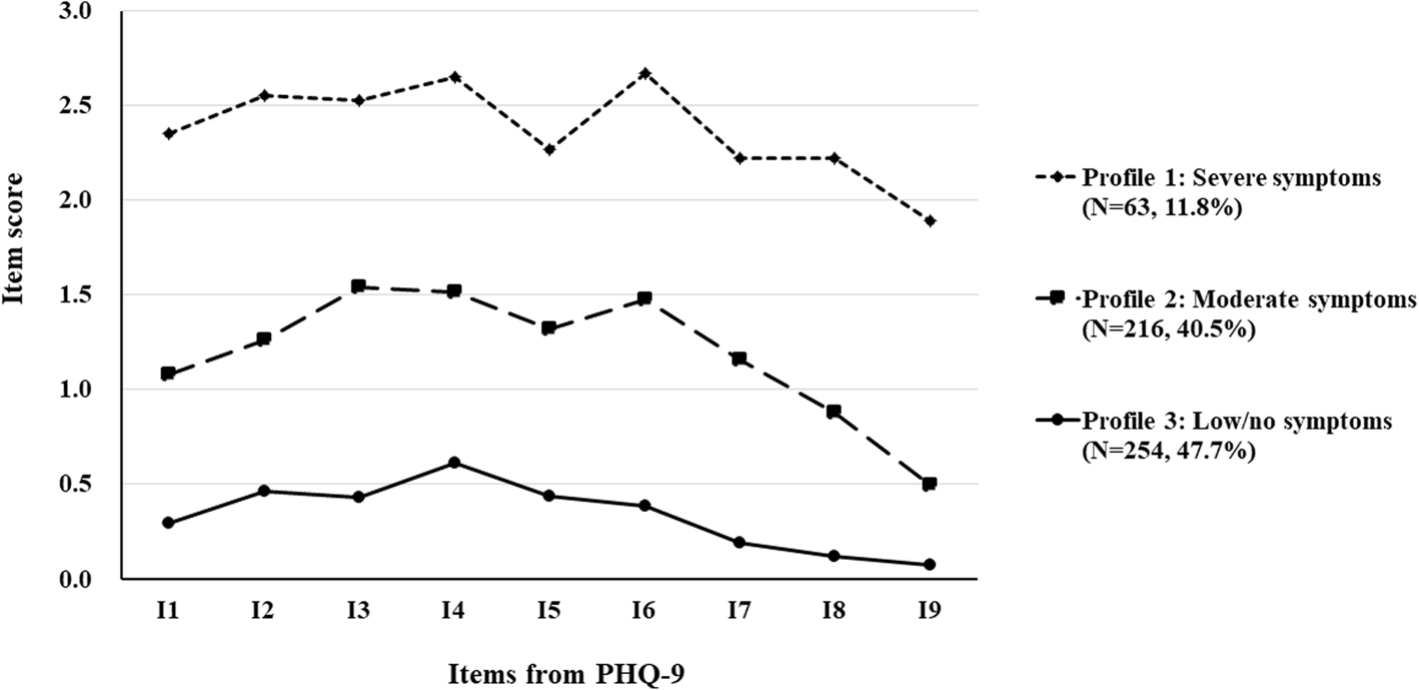
Mean depressive symptom cluster scores in the three latent profiles

The latent profile plot for our sample is presented in Fig. 1. Profile 1 comprised approximately 11.8% of participants (*N* = 63). PLWHs in Profile 1 were most likely to experience all depressive symptoms, and this profile was labeled as “severe symptoms”. Profile 3 comprised 47.7% of participants (*N* = 254) and included PLWHs who suffer less episodes of depressive symptoms. This profile was labeled as “low/no symptoms”. Additionally, profile 2 comprised approximately 40.5% of participants (*N* = 216) and included PLWHs with a high frequency of some of the depressive symptoms compared to the PLWHs reported in profile 3, while lower than those belonging to profile 1. The PLWHs in profile 2 showed elevations on Item 3 (sleep disturbance), Item 4 (fatigue or loss of energy), and Item 6 (feelings of worthlessness or excessive or inappropriate guilt) compared to other items. This profile was labeled as “moderate symptoms”.

### Univariate analysis

Table 1 also shows the relationships between sample characteristics variables and profiles of depressive symptoms among PLWHs. Participants that are prisoners, those with severe family dysfunction, lower resilience, or greater number of childhood trauma were more frequently categorized into the “severe symptoms” profile. Age and duration of HIV infection were found to vary significantly among three depressive symptoms profiles. PLWHs’ route of infection correlated with profiles of depressive symptoms borderline. In addition, there is no significant difference in sex, ethnicity, marital status, and residence location between three profiles.

### Regression analysis

The “low/no symptoms” profile (Profile 3) was the most prevalent profile in the present study. Thus, we used it as the reference class. Significance factors from the univariate analysis are included in the ordered logistic regression analysis. Results of multinomial logistic regression analysis of factors associated with depressive symptoms subtypes are listed in Table 3.

**Table 3:** Psychosocial factors associated with heterogeneity of depressive symptoms

Variables		Profile 1 Severe disturbance	Profile 2 Moderate disturbance
Participant type	Prisoners	Ref	Ref
	Outpatients	1.82(0.58,5.68)	2.45(0.80,7.44)
Age (years),	-	0.98(0.94,1.03)	0.99(0.95,1.03)
Education (years),	-	0.93(0.81,1.07)	0.92(0.81,1.05)
Duration of HIV infection (months)	-	1.00(0.99,1.01)	1.00(0.99,1.01)
Route of infection, [N(%)]	Sexuality	Ref	Ref
	Blood transfusion	1.06(0.46,2.47)	1.10(0.49,2.50)
	Needle sharing/ take drugs	0.88(0.31,2.48)	0.55(0.20,1.48)
	Unknown	0.37(0.01,9.94)	0.94(0.08,11.01)
Family function	Good	Ref	Ref
	Moderate dysfunction	2.74(1.09,6.89)^*^	2.45(0.95,6.34)
	Severe dysfunction	7.60(2.91,19.83)^***^	4.39(1.60,12.02)^**^
Resilience	High	Ref	Ref
	Medium	4.67(2.02,10.84)^***^	2.16(0.59,7.95)
	Low	19.87(5.70,69.28)^***^	2.86(1.30,6.25)^**^
Childhood trauma, [No as Ref.]	Emotional abuse	10.69(1.89,60.34)^**^	2.39(0.76,7.48)
	Physical abuse	2.20(0.66,7.35)	1.37(0.46,4.01)
	Sexual abuse	2.14(0.99,4.65)	1.42(0.68,2.94)
	Emotional neglect	3.97(1.50,10.53)^**^	2.78(1.09,7.12)^*^
	Physical neglect	1.44(0.62,3.31)	0.94(0.42,2.12)

The reference category for the dependent variables was the “Profile 3, High resistance”

*OR* Odds ratio, *CI* Confidence interval

^*^p < 0.05, ^**^p < 0.01, ^***^p < 0.001; Bold type indicates a significant odds ratio

Moderate (OR = 2.74, 95%CI = 1.09 ~ 6.89) and severe (OR = 7.60, 95%CI = 2.91 ~ 19.83) family dysfunction was found to predict the “severe symptoms” profile relative to the “low/no symptoms” profile. Resilience was a strong predictor of membership in the “severe symptoms” profile. More specifically, individuals with medium (OR = 4.67, 95%CI = 2.02 ~ 10.84) and low resilience (OR = 19.87, 95%CI = 5.70 ~ 69.28) being nearly 5 and 20 times more likely to be a member of the “severe symptoms” profile than the “low/no symptoms” profile, respectively. PLWHs experiencing emotion abuse (OR = 10.69, 95%CI = 1.89 ~ 60.34) and emotion neglect (OR = 3.97, 95%CI = 1.50 ~ 10.53) were more likely to be classified into the “severe symptoms” profile, in relation to the “low/no symptoms” profile. Moreover, compared to the “low/no symptoms” profile, severe family dysfunction (OR = 4.39, 95%CI = 1.60 ~ 12.02), low resilience (OR = 2.86, 95%CI = 1.30 ~ 6.25), and experience of emotional neglect (OR = 2.78; 95% CI = 1.09 ~ 7.12) indicated a higher likelihood of being classified in the “moderate symptoms” profile.

## Discussion

The present study was designed to identify profiles of depressive symptoms among Chinese PLWHs. Three distinguished profiles were identified according to frequency of depressive symptoms using LPA analysis: “severe symptoms”, “moderate symptoms”, and “low/no symptoms”. Additionally, family function, resilience, and childhood trauma were found to be differentiated factors for these profiles.

Three latent profiles of depressive symptoms among PLWHs were identified in this study, which is similar to the results of a previous study with a small sample [42]. Norcini and colleagues explored the heterogeneity of inflammation-related depression in 102 HIV-patients using LCA that uses binary indicators (symptom absence or presence), showing three classes: “severe symptoms”, “moderate symptoms”, and “low/no symptoms” [42]. LPA is a type of LCA that uses continuous indicators (scores) of symptom severity rather than binary indicators, which can provide more refined assessment of symptom profiles of information. In the current sample, the profile of PLWHs that showed high frequencies on all depressive symptoms was labeled as the “severe symptoms” profile. This profile accounted for the lowest proportion (11.8%) of participants, which is similar to recent research [43] showing that the prevalence of clinical level of depressive symptoms was 13.3% measured by PHQ-9 at a cut-off point of 10. Such PLWHs in “severe symptoms” profile, symptoms of fatigue or loss of energy and feelings of worthlessness or excessive or inappropriate guilt were the most prominent. Consistent with previous studies, PLWHs reported high rates of feeling of failure and worthless (52.8%) [44]. Jong et al. (2021) have also found that fatigue has a high prevalence among PLWHs and is strongly associated with depressive symptoms [45]. The percentage of PLWHs in the “moderate symptoms” profile included 40.5% of the participants in our total sample. The members of this profile reported mild/moderate depressive symptoms, for which the most prominent symptom is sleep disturbance. PLWHs commonly existed sleep disruptions and poor sleep-induced fatigue [46], which consistently have been shown to induce depressive symptoms for PLWHs [47]. Furthermore, our study identified that nearly half of PLWHs fell within the “low/no symptoms” profile (47.7%), which is similar with the result of a systematic review and meta-analysis for PLWH in China (49.2%) [3]. This result indicated that the many PLWHs could cope well to HIV infection. In China, the implementation of the “Four Frees and One Care” policy [48] not only allows HIV/AIDs patients to enjoy free antiretroviral therapy (ART), but also to receives government support in education and employment. These initiatives are beneficial to maintain and promote the physical and mental health of PLWHs.

In the logistic model, PLWHs living in a severe family dysfunction were above 7 times and 4 times more likely to be found in the “severe symptoms” profile and “moderate symptoms” profile than those experienced good family function. A great volume of literature has consistently asserted that family function as a protective factor for depressive symptoms [18, 49]. Family function is a multi-faceted construct that primarily focuses on family cohesion and family adaptability [50], which thought to be protective factors in coping with stressor [51, 52], thereby reduced individual’ depressive symptoms. Meanwhile, low resilience seemed to be the strongest predictive factor for development of depressive symptoms. Resilience, as a dynamic course and drives a person to grow in the face of adversity [53]. Resilience is proposed as a potential factor to ameliorate negative emotions and help maintain well-being [54]. Our results also indicate the possible impact of childhood trauma on the development of depressive symptoms among PLWHs. Specifically, emotional abuse and neglect, but not sexual abuse, physical abuse and neglect are predictive of depressive symptoms in PLWHs. Our findings fit with previous results suggesting childhood emotional trauma plays a more important role in depression than other types of childhood trauma [55, 56]. As reported in previous studies [57, 58], emotional abuse is associated with emotion dysregulation, while emotional neglect is associated with deficiency in adaptive emotion regulation. Furthermore, based on social learning theory, individuals who are emotionally neglected may not be able to learn adaptive emotion regulation strategies through caregiver modeling, which predisposes them to depression in adulthood [59].

Our findings suggest that therapeutic interventions targeting depressive symptoms may benefit from a tailored approach that considers individual symptom patterns of depression. For example, PLWHs in profile 1 (severe symptoms) have severe overall symptoms and therefore require prompt clinical treatment to help restore good emotional functioning. Meanwhile, mild/moderate depressive symptoms were present in PLWH in profile 2 (moderate symptoms), with sleep disturbances being the most prominent. Therefore, interventions can be carried out to address sleep problems (e.g., cognitive behavioral therapy [60], mindfulness-based interventions [61]), with the aims to improve not only sleep problems but in turn enhance PLWHs’ mood. Moreover, influential factors should also be taken into consideration for effective psychosocial intervention for PLWHs. Empirical studies have shown the effectiveness of multiple family therapies in improvement in family function, and thereby amelioration of depressive symptoms [62]. Starting from resilience or trauma may be an effective way to protect mental health of HIV/AIDs patients, such as the Improving AIDS Care after Trauma (ImpACT) [63] and Resiliency-based intervention [64].

Despite all the relevant findings, several limitations of the current study should be noted. First, our measures of depressive symptoms and other psychological factors relied on self-report questionnaires, which might be influenced by reporting bias caused by recollection inaccuracy and individuals’ own psychiatric states. Meanwhile, although the internal consistency of PHQ-9 in the current sample was high (α = 0.92), the validity of the Chinese version of PHQ-9 in PLWHs has not been fully tested and therefore needs to be interpreted with caution. Second, the data were collected in only one province of China, which is uncertain whether our findings could be generalized to all PLWHs to other regions of China. Thus, future studies would benefit from examining depressive symptoms in samples that are more representative of the PLWH in China. Third, some confounding factors associated with PLWHs’ depressive symptoms were not considered, such as HIV-related stigma [65]. Finally, PLWH sample of this study was composed of two separate groups, namely outpatients and prisoners. Significant differences in some of the socio-demographic characteristics and HIV-related factors (e.g., age, sex, duration of HIV infection) found to exist between these two groups in the previous study [20]. In addition, the prisoners included in this study were incarcerated in specific prisons, and social isolation may have a potential impact on the patients’ depressive symptoms. Therefore, results need to be interpreted with caution.

## Conclusion

In summary, we provide evidence of distinct profiles for depressive symptoms in a sample of Chinese PLWHs, which were defined as the “severe symptoms”, “moderate symptoms”, and “low/no symptoms”, respectively. Family function and resilience served as strong protective factors against depressive symptoms, while childhood trauma, especially emotional abuse and neglect contributed as risk factors. These factors should also be taken into consideration for effective psychosocial intervention for PLWHs.

## Data Availability

The datasets used and/or analyzed during the current study are available from the corresponding author (Xuan Ouyang: ouyangxuan@csu.edu.cn) on reasonable request.
